# Self‐administered biofeedback treatment app for pediatric migraine: A randomized pilot study

**DOI:** 10.1002/brb3.1974

**Published:** 2020-12-08

**Authors:** Anker Stubberud, Mattias Linde, Eiliv Brenner, Martin Heier, Alexander Olsen, Anne Hege Aamodt, Gøril B. Gravdahl, Erling Tronvik

**Affiliations:** ^1^ Department of Neuromedicine and Movement Science NTNU Norwegian University of Science and Technology Trondheim Norway; ^2^ Norwegian Advisory Unit on Headaches, Department of Neurology St. Olavs Hospital Trondheim Norway; ^3^ Department of Clinical Neuroscience for Children Oslo University Hospital Oslo Norway; ^4^ Department of Psychology NTNU Norwegian University of Science and Technology Trondheim Norway; ^5^ Department of Physical Medicine and Rehabilitation St. Olavs Hospital Trondheim Norway; ^6^ Department of Neurology Oslo University Hospital Oslo Norway

**Keywords:** adolescent, headache, mHealth, smartphone, wearables

## Abstract

**Objective:**

To investigate the effect size, safety, and tolerability of a therapist‐independent biofeedback treatment app among adolescent with migraine.

**Materials and Methods:**

This was a prospective, 3:1 ratio randomized, sham‐controlled, double‐blind, pilot study with 16 adolescents diagnosed with migraine randomized to eight weeks of biofeedback treatment (*n* = 12) or sham biofeedback (*n* = 4), carried out at two university hospitals in Norway. The prespecified and primary objective of the study was to observe changes in outcomes within the active treatment group. The sham control group was included in a minor ratio primarily to evaluate its feasibility. The primary outcome was change in headache frequency. A modified intention to treat analysis was performed, including participants completing at least seven biofeedback sessions in weeks 1–4 (*n* = 12 vs. *n* = 4) and weeks 5–8 (*n* = 7 vs. *n* = 2).

**Results:**

Adherence was poor with 40% (136/336) of planned biofeedback sessions completed during weeks 5–8. Within the biofeedback group, a not statistically significant reduction in headache frequency was observed at weeks 1–4 (2.92 days/month, 95% CI −1.00 to 6.84, *p* = .145) and weeks 5–8 (1.85 days/month, 95% CI −2.01 to 5.72, *p* = .395). The biofeedback group experienced a median of one fewer headache days/month versus sham that did not reach significance (95% CI −4.0 to 9.0, *p* = .760).

**Conclusions:**

We observed a small reduction in headache frequency in the active treatment group. Findings were likely undermined by low adherence and underpowered analyses but indicate that a therapist‐independent biofeedback treatment app has the potential to be an effective, tolerable, and inexpensive treatment option.

## Graphical abstract

Biofeedback is an effective migraine treatment; however, a completely therapist‐independent smartphone administered treatment for children and adolescents has not been investigated. In this randomized controlled trial, we show that smartphone biofeedback treatment has the potential to be an effective in pediatric migraine, reducing migraine frequency by approximately 30%.

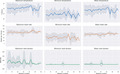

## 1 INTRODUCTION

Pediatric migraine is highly prevalent and associated with substantial deterioration of social functioning and mental health (Krogh et al., 2015; Wober‐Bingol, 2013). Those in need of prophylactic treatment are faced with few viable options as most pharmacological prophylaxes have limited efficacy or unacceptable adverse effects (El‐Chammas et al., 2013; Oskoui et al., 2019; Powers et al., 2017; Termine et al., 2011). However, behavioral therapies, and especially biofeedback, appears to be a suitable treatment option for children and adolescents with headache (Fisher et al., 2018; Stubberud et al., 2016; Trautmann et al., 2006).

During biofeedback, individuals learn to voluntarily modify their bodily reactions through feedback from their own physiological processes. Commonly used physiological parameters are peripheral skin temperature, frontal or trapezius muscle surface electromyographic voltage (SEMG) and blood‐volume‐pulse (Schwartz & Andrasik, 2017). Traditionally, biofeedback is delivered in a clinic with suited measurement devices and a trained therapist. The therapist assists with the technical use of the measurement devices and provides the user with insights on how to interpret and modify the physiological parameters. Regular biofeedback training reduces central nervous system arousal, renders individuals more resilient to environmental stressors, and ultimately lowers migraine burden (Lehrer & Eddie, 2013; Siniatchkin et al., 2000). Unfortunately, the time‐consuming and cumbersome nature of the treatment has resulted in limited population coverage (Penzien et al., 2015).

The rapidly growing use of wearables and smartphone mobile applications (apps) for medical purposes (mHealth) allows for simpler ways of administering biofeedback (Stubberud & Linde, 2018). mHealth poses many potential areas of application in headache medicine, but most of these remains to be explored (Lalloo et al., 2015). Specifically, no app‐based biofeedback as prophylaxis for migraine in children and adolescent exists (Minen et al., 2016; Mosadeghi‐Nik et al., 2016). To start filling this gap of knowledge, we have validated the use of wearables suited for biofeedback and developed a self‐administered therapist‐independent biofeedback treatment app for pediatric migraine (Stubberud et al., 2018, 2020).

We hypothesized that treatment with a self‐administered biofeedback app could improve migraine burden among adolescents. Based on this we conducted a pilot study with a primary objective to investigate the effect size, safety, and tolerability of a biofeedback treatment app among adolescents with migraine. Secondly, we aimed to evaluate the feasibility of a sham biofeedback app and compare it to the active treatment. The study was intended to guide study design, choice of control group, and sample size calculation for future clinical trials.

## 2 METHODS

### 2.1 Study design and participants

The study was designed as a prospective, 3:1 ratio randomized, sham‐controlled, double‐blind, pilot study conducted at St. Olavs Hospital, Trondheim, Norway; and Oslo University Hospital, Oslo, Norway, with planned enrollment from January 2019 to June 2020. The study comprised a four‐week baseline period, followed by an eight‐week intervention period with either a biofeedback treatment app or a sham biofeedback app. No statistical power calculation was conducted prior to the study. We planned on recruiting 40 participants—to ensure at least 25 in the main intervention group—as this represents a number where further increase in precision with increased sample size is minimal (Johanson & Brooks, 2010). However, recruitment proceeded unexpectedly slow and was terminated prematurely in March 2020 due to the SARS‐CoV2 pandemic. Thus, 23 adolescents with migraine were recruited through repeated advertisements at pediatric clinics in the municipality, local mainstream media, social media patient groups, and the intranet at the university hospital in Trondheim. The study was approved by the regional ethics committee (Identifier: 2018/35) and the Norwegian Medicines Agency (Identifier: 18/12060‐9). The study was registered at clinicaltrials.gov (Identifier: NCT04106505). The study participants assented to partake in the study, and written informed consent was obtained from their guardians.

Inclusion criteria were (A) age between 12 and 18 years; (B) diagnosis of migraine with or without aura according to the international classification of headache disorders (ICHD‐3) (IHS, 2018); and (C) two to eight migraine attacks per month. Exclusion criteria were (A) participant not speaking Norwegian; (B) reduced sensibility, hearing or vision to a degree that impairs proper use of the app; (C) severe psychiatric or neurologic disease and; (D) participant currently using migraine prophylaxis. The rationale for excluding patients on prophylactic treatment or with more than eight migraine attacks per month was to primarily recruit treatment‐naïve patients as the proposed treatment is envisioned for widespread use in a primary‐care setting.

Eligible participants met with a consultant neurologist or pediatrician with headache expertise to confirm the migraine diagnosis. During baseline, participants were instructed to daily register maximal headache intensity, average headache intensity, functioning in daily activities, and abortive drug consumption in a paper headache diary. After a minimum 28‐day baseline period participants were randomly assigned to one of the two intervention groups by a computer‐generated block‐randomization list. In each block of four, participants had a 75% chance of being allocated to the biofeedback group and a 25% chance of being allocated to the sham group. Participants were asked to download the app and enter a 5‐digit number to unlock the app. The 5‐digit number was drawn by the enrolling physician sequentially from a list of 40 numbers. One random in every four numbers resulted in downloading a sham version of the app while the other three numbers resulted in downloading the proper biofeedback app. Both versions of the app looked alike and no pattern in the 5‐digit number or the randomization list could reveal which version of the app was given. This ensured blinding of participants, healthcare providers and investigators. Blinding of outcome assessors was not possible due to the 3:1 randomization ratio. Breaking of the randomization was performed only after follow‐up of the last participant, when the software developers revealed if the 5‐digit number corresponded to the biofeedback or sham version of the app.

During treatment, participants were asked to complete daily headache diary entries (the same questions as in the paper diary) and biofeedback sessions within the app. Participants were also encouraged to contact investigators with inquiries on how to use the equipment, report errors or shortcomings regarding both hardware and software, and take notes of any adverse events (AE) and report these to the researchers. Finally, participants met with one of the researchers at the end of the two‐month intervention period for evaluation, adverse event questioning, and to return the equipment.

### 2.2 Interventions

The active treatment arm comprised a self‐administered treatment app, including biofeedback training, instructions for self‐delivery, and a headache diary. The intervention was developed specifically for adolescents with migraine through a user‐involved iterative and incremental design cycle where the choice of biofeedback modalities, instructions and headache diary questions was based on feedback from adolescent users. Details on the development and validation of the intervention are provided in a paper describing the development and usability process (Stubberud et al., 2020). The app gave a push‐reminder to complete a headache diary entry and a biofeedback session of 10 min duration daily. Participants were allowed to set a custom daily timepoint for the reminder. The headache diary entry had to be completed to start a biofeedback session. Prior to commencing treatment, participants were given basic information on the rationale behind biofeedback treatment. Participants were instructed that the goal of the biofeedback sessions was to increase skin temperature and decrease heart rate and muscle tension. They were given some basic suggestions on how to complete this, such as lying down to “relax” or specifically focusing on a low heart rate, warm hands, or relaxing their neck and shoulder musculature. However, we aimed to keep the biofeedback training simplistic as possible, relying chiefly on the participants’ self‐achievable instrumentational conditioning from the biofeedback sensors. They were also given instructions on how to use the equipment and software, and how to complete a biofeedback session. Sham biofeedback was achieved by adding sine‐curve fluctuations to the correct feedback signal and thereby partly disrupting the true connection between the input of physiological parameters and the feedback. The looks and contents of the normal app and the sham app were completely similar. The only difference was the internal software algorithm, which was inaccessible to the user and investigators. All participants in both groups were given the same information and instructions. Participants were not instructed in relaxation techniques or stress management techniques. The intervention and sham are described in detail elsewhere (Stubberud et al., 2020).

The biofeedback source signal was produced by wireless wearable sensors measuring muscle tension, finger temperature, and heart rate. The bipolar surface electromyography sensor (NeckSensor™; EXPAIN AS, Oslo, Norway) was used for measuring SEMG muscle tension from the upper trapezius muscle fibers. The PASPORT Skin/Surface Temperature Thermistor Probe, PS‐2131 (Pasco, Roseville, CA, USA) was held between the index finger and thumb of the right hand to measure finger temperature. The MIO Fuse™ (Mio Global, Physical Enterprises) photoplethysmography heart rate wristband was used to measure heart rate over the dorsal aspect of the left wrist. Heart rate, rather than the more commonly used heart rate variability, was chosen as one of the physiological measurements as it is easily accessible and readily interpretable in a therapist‐independent setting. All sensors transmitted signals via Bluetooth^®^ Smart/4.0 to an iPhone^®^ 6 or newer.

### 2.3 Outcomes

The primary outcome was change in the frequency of headache days from baseline to end of treatment. Secondary outcomes were responder rate (more than 50% reduction in headache frequency); change in maximal and average pain intensity recorded on a ordinal 4‐point scale (0 = no headache, 3 = severe headache); change in functioning in daily activities recorded on a ordinal 4‐point scale (0 = no problems with daily activities, 3 = severe problems with daily activities); change in number of days with abortive drug consumption; and AEs. Participants were asked specifically to report any skin reactions, nausea and dizziness, and any additional AEs were recorded.

Headache‐related functioning in daily activities and average pain intensity was not prespecified in the protocol and was included in the headache diary prior to enrollment as per trial guideline recommendations (Tfelt‐Hansen et al., 2012). While the prespecified and primary objective of this pilot study was to observe the outcomes *within* the biofeedback group only, we also conducted post hoc comparative analyses of outcomes *between* the two groups. We also conducted a second post hoc response rate analysis, changing the response threshold to 30% or greater reduction in headache frequency. This was deemed suitable as none of the participants had a baseline headache frequency below 3 days per week. Finally, we included a post hoc analysis of mean change in biofeedback physiological parameters from the start to the end of sessions. Apart from these post hoc analysis alterations, the trial was conducted according to the original protocol.

### 2.4 Data management and statistical analyses

This is the first analysis of data collected in this study. The analysis was conducted after all patients completed the final visit or terminated participation in order to maintain blinding. At all visits, data were collected and recorded on a paper clinical report form. Paper headache diaries were collected at the end of the baseline period. Baseline headache data was calculated from the last 28 days of the baseline period. The SEMG, temperature, and heart rate measurements for each biofeedback session, along with headache diary data were transferred daily to a secure database. A priori we planned to conduct an intention to treat (ITT) analysis of all randomized patients comparing baseline data to the last 28 days (weeks 5–8) of treatment. However, because several participants did not complete any biofeedback sessions during weeks 5–8 (and thus did not receive treatment and had no headache diary entries) and to avoid imputing data, we conducted a modified ITT (mITT) analysis. To be included in the mITT analysis participants were required to have completed at least 7 of the planned 28 headache diary entries in weeks 5–8. Because all participants completed at least seven biofeedback sessions and headache diary entries during weeks 1–4, we also included an analysis comparing baseline to weeks 1–4. We used only available data in the analyses with no imputation of data.

Adherence was evaluated as the proportion of completed treatment sessions and headache diary entries (out of 56 planned sessions in the eight weeks following treatment start). The mean SEMG, temperature, and heart rate measurements from the first and last minute of sessions lasting more than five minutes were summarized. We also calculated the median of the ten largest values, the median of the ten smallest values, and the overall mean for the SEMG, temperature, and heart rate recordings from each biofeedback session. The latter data were visualized by plotting the average value across all individuals for each completed session with a moving average smoothing function with a window width of three sessions.

Data were reported as means, standard deviations (*SD*), medians, and interquartile ranges (IQR). Within‐group changes were analyzed with a two‐tailed Wilcoxon signed rank test and summarized with mean differences (MD) with 95% confidence intervals (CI). A two‐tailed Mann–Whitney U test was used to compare changes in outcomes between the two groups and median effect estimates with 95% CI were produced with the Hodges‐Lehman estimator. Finally, to analyze for changes in the physiological measurements between the start and end of biofeedback sessions we performed a two‐tailed paired *t*‐test and summarized the findings using MDs with 95% CI. Normality assumptions were based on visual inspection of histograms. *p*‐values were evaluated at the .05 significance level.

All statistical analyses and figures were made with Python (v.3.7.7, Python Software Foundation) with the following open‐source packages: matplotlib v.3.2.1, NumPy v.1.18.2, pandas v.0.20.3, PyNonpar v.0.2.0, scipy v.1.4.1, and seaborn v.0.10.0.

## 3 RESULTS

**FIGURE 1:**
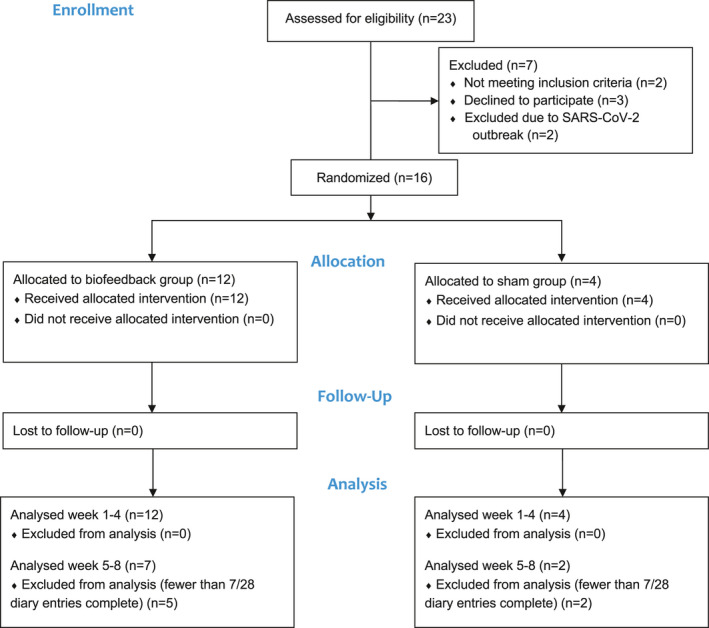
CONSORT flow diagram

Twenty‐three participants were recruited, 18 from St. Olavs University Hospital and five from Oslo University Hospital. Seven participants were excluded or dropped out during the baseline period, and 16 patients were randomized (reasons for exclusion in Figure 1). Twelve participants were randomized to the biofeedback group and four were randomized to the sham group. All randomized participants were analyzed at weeks 1–4. Seven participants in the biofeedback group and two in the sham group were analyzed at weeks 5–8. Participant demographics are provided in Table 1. Overall, the proportion 79% (353/448) of planned headache diary entries were completed during weeks 1–4 and 48% (214/448) were completed during weeks 5–8. In the biofeedback group, 58% (196/336) of planned biofeedback sessions were completed during weeks 1–4 and 40% (136/366) were completed during weeks 5–8. In the sham group, 65% (73/112) of planned biofeedback sessions were completed during weeks 1–4 and 30% (34/112) were completed during weeks 5–8. Three out of four participants allocated to the sham group believed they received sham treatment, whereas one of the participants in the biofeedback group believed they received sham treatment.

**TABLE 1:** Participant demographics

	Biofeedback group (*n* = 12)	Sham group (*n* = 4)
Age, mean ± *SD* (range)	15 ± 2 (13–18)	14 ± 2 (12–16)
Female, *n* (%)	10 (83%)	1 (25%)
Migraine aura, *n* (%)	9 (75%)	2 (50%)
Other headache disorders		
TTH, *n* (%)	8 (67%)	3 (75%)
MOH, *n* (%)	1 (8%)	1 (25%)
Tried triptans, *n* (%)	9 (75%)	3 (75%)
Tried migraine pharmacoprophylaxis, *n* (%)	3 (25%)	1 (25%)

### 3.1 Outcomes in the biofeedback group

A not statistically significant mean reduction in headache frequency of 2.9 days/month (95% CI −1.0 to 6.8, *p* = .145) was reported during weeks 1–4. A not statistically significant mean reduction in headache frequency of 1.9 days/month (95% CI −2.0 to 5.7, *p* = .395) was reported during weeks 5–8. No statistically significant changes in maximal headache intensity, average headache intensity, headache‐related daily functioning, or abortive drug consumption were observed within the biofeedback group (Table 2). In the biofeedback group, 4 out of 12 (33%) participants were considered responders at weeks 1–4, and 2 out of 7 (29%) participants were considered responders at weeks 5–8. Moreover, 9 out of 12 (75%) participants experienced ≥30% reduction in headache frequency during weeks 1–4 and 2/7 (29%) experienced a ≥30% reduction in headache frequency during weeks 5–8.

**TABLE 2:** Median and mean estimates of headache outcomes at baseline, weeks 1–4 and weeks 5–8 *within* the biofeedback group

		Baseline (*n* = 12)	Week 1–4 (*n* = 12)	Week 5–8 (*n* = 7)	Baseline versus weeks 1–4, MD (95% CI); *p*‐value	Baseline versus weeks 5–8, MD (95% CI); *p*‐value
Headache frequency	Median (IQR)	10.0 (7.0–14.0)	8.0 (4.5–14.0)	8.0 (5.5–14.0)	−2.9 (95% CI −6.8 to 1.0); *p* = .145	−1.9 (95% CI −5.7 to 2.0); *p = *.395
	Mean (*SD*)	11.2 (5.5)	8.2 (6.0)	9.6 (7.3)		
Maximum intensity	Median (IQR)	1.7 (1.7–1.9)	1.8 (1.4–1.9)	2.0 (1.7–1.9)	−0.3 (95% CI −1.0 to 0.4); *p = *.754	−0.1 (95% CI −0.8 to 0.6); *p = *.735
	Mean (*SD*)	1.9 (0.5)	1.5 (0.8)	1.7 (0.8)		
Average intensity	Median (IQR)	1.5 (1.4–1.7)	1.4 (1.2–1.7)	1.6 (1.2–1.7)	−0.2 (95% CI −0.7 to 0.3); *p = *.347	−0.1 (95% CI −0.9 to 0.6); *p = *.735
	Mean (*SD*)	1.5 (0.3)	1.2 (0.7)	1.4 (0.7)		
Daily functioning	Median (IQR)	1.0 (1.0–1.0)	1.0 (1.0–1.0)	1.0 (1.0–1.0)	−0.2 (95% CI −0.4 to 0.1); *p = *.157	−0.1 (95% CI −0.5 to 0.2); *p = *.317
	Mean (*SD*)	1.0 (0.0)	0.8 (0.4)	0.9 (0.4)		
Abortive drug consumption	Median (IQR)	6.5 (2.8–10.0)	3.0 (1.0–10.0)	7.0 (4.0–10.0)	−3.1 (95% CI −6.9 to 0.7); *p = *.092	−1.9 (95% CI −6.8 to 3.1); *p = *.446
	Mean (*SD*)	7.2 (6.0)	4.2 (4.2)	6.7 (4.6)		

Note

The two rightmost columns show the mean difference with 95% confidence intervals and *p*‐values of a two‐tailed Wilcoxon signed rank test comparing outcomes at weeks 1–4 and weeks 5–8 versus baseline.

Abbreviations: CI, confidence interval; IQR, interquartile range; MD, mean difference; *SD*, standard deviation.

### 3.2 Between‐group comparisons

No statistically significant difference in change in headache frequency between the two groups was reported during weeks 1–4 (0.5 headache days/month, 95% CI −9.0 to 16.0, *p* > .999), and weeks 5–8 (−1.0 headache days/month, 95% CI −9.0 to 4.0, *p = *.760). There was no statistically significant difference between the two groups in any of the secondary outcomes (Table 3).

**TABLE 3:** Changes in headache outcomes in the biofeedback group vs. sham group at weeks 1–4 and weeks 5–8

	Group	Baseline, median (IQR)	Median change score at weeks 1–4 (IQR)	Hodges‐Lehmann estimate of effect size week 0 vs. week 4 (95% CI); *p*‐value
mITT between‐group comparison baseline vs. weeks 1–4 (BFB *n* = 12; sham *n* = 4)				
Headache frequency	BFB	10.0 (7.0 to 14.0)	−4.0 (−6.2 to −3.8)	0.5 (95% CI −9.0 to 16.0); <.999
	Sham	12.5 (8.2 to 18.2)	−3.0 (−9.8 to 1.2)	
Maximum intensity	BFB	1.7 (1.7 to 1.9)	−0.1 (−0.3 to 0.3)	0.1 (95% CI −0.6 to 0.6); .585
	Sham	2.2 (2.0 to 2.3)	−0.2 (−0.3 to −0.1)	
Average intensity	BFB	1.5 (1.4 to 1.7)	−0.1 (−0.3 to 0.1)	−0.2 (95% CI −0.8 to 0.3); .303
	Sham	1.5 (1.3 to 1.8)	0.1 (−0.0 to 0.2)	
Daily functioning	BFB	1.0 (1.0 to 1.0)	0.0 (0.0 to 0.0)	0.0 (95% CI 0.0 to 0.0); .46
	Sham	1.0 (1.0 to 1.0)	0.0 (0.0 to 0.0)	
Abortive drug consumption	BFB	6.5 (2.8 to 10.0)	−4.0 (−7.5 to 0.5)	−5.0 (95% CI −10.0 to 3.0); .301
	Sham	3.0 (1.0 to 5.0)	−0.5 (−1.0 to 1.2)	

	Group	Baseline, median (IQR)	Median change score at weeks 5–8 (IQR)	Hodges‐Lehmann estimate of effect size week 0 vs. week 8 (95% CI); *p*‐value
mITT between‐group comparison baseline vs. weeks 5–8 (BFB *n* = 7; sham *n* = 2)				
Headache frequency	BFB	9.0 (7.0 to 15.0)	−1.0 (−4.0 to 0.5)	−1.0 (95% CI −9.0 to 4.0); .760
	Sham	7.5 (6.8 to 8.2)	0.0 (−0.5 to 0.5)	
Maximum intensity	BFB	1.7 (1.7 to 1.9)	0.1 (−0.1 to 0.4)	0.2 (95% CI −1.6 to 0.8); .883
	Sham	2.3 (2.3 to 2.3)	−0.1 (−0.1 to −0.1)	
Average intensity	BFB	1.5 (1.5 to 1.7)	−0.2 (−0.3 to 0.3)	−0.4 (95% CI −2.0 to 0.6); .464
	Sham	1.8 (1.8 to 1.9)	0.3 (0.2 to 0.3)	
Daily functioning	BFB	1.0 (1.0 to 1.0)	0.0 (0.0 to 0.0)	0.0 (95% CI −1.0 to 0.0); .789
	Sham	1.0 (1.0 to 1.0)	0.0 (0.0 to 0.0)	
Abortive drug consumption	BFB	7.0 (4.0 to 13.0)	−1.0 (−5.5 to 2.5)	−2.0 (95% CI −12.0 to 4.0); .769
	Sham	5.0 (5.0 to 5.0)	1.0 (0.5 to 1.5)	

Note

Note that negative values in the rightmost column indicates a favor toward the biofeedback group.

Abbreviations: BFB, biofeedback; CI, confidence interval; IQR, interquartile range.

### 3.3 Physiological measurements

**FIGURE 2:**
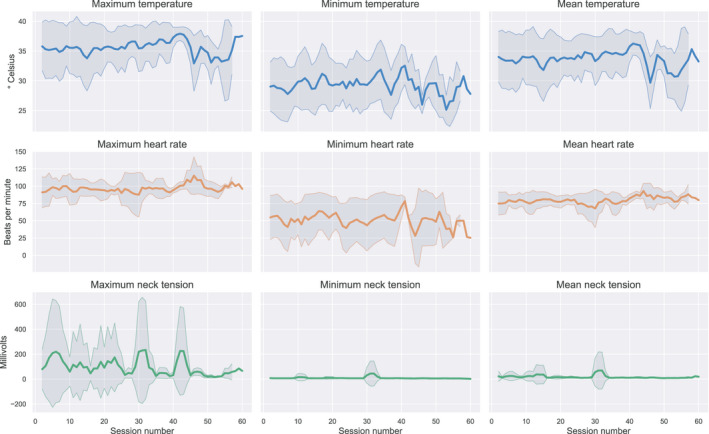
Visualization of raw physiological data over the course of all biofeedback sessions for patients in the verum group. The thick lines represent mean raw physiological value averaged over a moving window of three sessions. The shaded gray area represents the corresponding moving average of one standard deviation from the mean. Note the slight increasing trend in finger temperature, and slight decreasing trend in minimum heart rate and maximum muscle tension up to session 40. The number of participants completing more than 40 sessions was low, thus yielding the highly variable trends in session 40–60

Table 4 summarizes the physiological measurements at the biofeedback session start and session end in the biofeedback group. Within sessions, participants achieved a statistically significant increase in finger temperature (4.4° Celsius; 95% CI 4.0 to 4.8; *p* < .001), increase in heart rate (5.6 beats per minute; 95% CI 3.3 to 8.0; *p* < .001), and reduction in SEMG voltage (15.1 millivolts; 95% CI 6.6 to 23.7; *p* = .0006). Across all sessions, we observed a slightly increasing trend in maximum finger temperature, and a slightly decreasing trend in minimum heart rate and maximum muscle tension. Figure 2 visualizes the SEMG, temperature and heart rate measurements across all sessions in the biofeedback group.

**TABLE 4:** Physiological measurements in the biofeedback sessions

		Two first sessions		Two middle sessions		Two last sessions	
		Verum	Sham	Verum	Sham	Verum	Sham
Peripheral skin temperature, °Celsius (*SD*)	Session start	30.7 (4.0)	31.5 (3.2)	31.6 (3.9)	32.6 (3.5)	32.8 (3.4)	33.3 (3.3)
	Session end	36.3 (4.1)	37.5 (1.1)	36.1 (3.9)	37.5 (2.0)	37.6 (1.6)	36.6 (2.6)
Heart rate, bpm (*SD*)	Session start	71.0 (24.7)	63.3 (19.6)	77.5 (17.5)	65.0 (16.6)	74.0 (20.7)	73.6 (14.5)
	Session end	81.7 (10.5)	72.8 (13.5)	80.9 (8.6)	69.0 (15.3)	79.0 (6.0)	70.2 (11.3)
Trapezius SEMG voltage, mV (*SD*)	Session start	15.1 (18.2)	19.4 (18.6)	19.5 (32.5)	18.5 (15.8)	20.5 (32.6)	19.8 (17.9)
	Session end	8.8 (2.2)	8.0 (0.8)	8.5 (1.6)	10.6 (7.3)	17.0 (37.1)	16.6 (18.9)

Note

The table shows the mean physiological measurements of the first and last minute of sessions with a duration of at least five minutes. Because participants completed different number of sessions, we compared the average of the two first sessions with the average of the two middle sessions and the two last sessions. Note that while there is a slight increase in end session temperature from the two first sessions to the two last sessions, the amplitude of within‐session change is diminished throughout sessions. Moreover, this trend appears to be comparable in the verum and sham groups.

Abbreviations: bpm, beats per minute; mV, millivolts; *SD*, standard deviation; SEMG, surface electromyography.

### 3.4 Safety and tolerability

One single AE was reported by a participant experiencing a mild skin rash related to the SEMG electrode patch. The rash lasted for a week without treatment. None of the other prespecified AEs were reported.

## 4 DISCUSSION

### 4.1 Principal findings

To the best of our knowledge, this is the first trial investigating the use of a mHealth biofeedback intervention designed specifically for migraine in adolescents. Overall, the study suffered from attrition, difficulties in the recruitment process and prematurely terminated data collection due to the SARS‐CoV‐2 pandemic. No statistically significant reduction in headache frequency in the active treatment group or superiority over sham was observed. Still, several patients experienced a meaningful reduction in headache frequency, and the intervention was nearly free of AEs. The findings should be used as guidance in planning and designing future studies of therapist‐independent app‐based biofeedback treatment.

### 4.2 Interpretation

Meta‐analyses have found that biofeedback is effective in treating pediatric migraine, at least when compared to a waiting list control (Fisher et al., 2018; Stubberud et al., 2016). Treatment effect is typically in the range of 35%–50% reduction in headache frequency (Penzien et al., 2002). In this study, we observed an approximate 20% reduction in headache frequency, which is lower than the typical treatment effect. Several factors may contribute to understanding why we observed a limited treatment effect that was not statistically significant.

Firstly, the nature of the biofeedback intervention used in the present study was quite different from traditional biofeedback. Usually, the treatment is administered as a “treatment package” with regular therapist contact sessions and combined with adjunctive behavioral therapies such as relaxation and stress management. The therapist aids the user in achieving the “correct” self‐control, and the treatment package promotes several of the nonspecific effects seen with biofeedback, such as expectancy, conditioning, and regular contact and procedural repetitions (Autret et al., 2012). In the present study, participants were given a very minimalistic intervention, only consisting of a brief introduction to the concept of biofeedback and brief instructions on how to use the equipment and perform a session. Thereafter, learning self‐control was entirely based on operant conditioning from the feedback instruments. Participants appeared to quickly learn to increase temperature and lower muscle tension within biofeedback sessions. However, there was no clearly evident improvement across sessions, and we also observed a paradoxical increase in heart rate within sessions. A real‐world therapist could potentially have helped to modulate the self‐control toward the assumed “correct” state, which is hypothesized to predict positive outcomes (Lisspers et al., 1992). Moreover, the absence of therapist contact and adjunctive therapies may have led a reduction in the nonspecific effects, further explaining the limited treatment effect (Autret et al., 2012). Even though previous studies have found that limited‐contact biofeedback may be as efficacious as traditional biofeedback (Burke & Andrasik, 1989; Guarnieri & Blanchard, 1990; Scharff et al., 2002), these still employed much more comprehensive treatment packages than was used in the present study. On the other hand, a more similar study, investigating the effect of one single biofeedback training session, followed by self‐directed practice sessions observed a reduction in headache frequency from 12.9 to 9.7 days/months, which is more in line with our findings (Powers et al., 2001).

Secondly, the adherence rate to biofeedback treatment in the present study was low, potentially resulting in reduced treatment effects. A systematic review found that the adherence to behavioral interventions among children varied between 52% to 86% (Ramsey et al., 2014). This is superior to what we observed, especially in weeks 5–8. There are no clear estimates of how much adherence influences treatment outcome, but lower adherence is believed to undermine the efficacy of behavioral interventions (Gewirtz & Minen, 2019). A study of app‐based progressive muscle relaxation as a prophylactic treatment for migraine in adults found that highly adherent users (defined as two or more session per week) had a significantly greater reduction in headache frequency than users with low adherence (Minen et al., 2019). This supports our findings, where the reduction in headache frequency in the biofeedback group was greatest in weeks 1–4, the period where adherence was the highest.

Thirdly, the limited data in the study likely means that there was insufficient power to detect a statistically significant change in headache frequency. A priori we planned to recruit 40 participants, to ensure at least 25 in the biofeedback group. This is twice the number that was allocated to biofeedback treatment, and a larger sample size may indeed have revealed a statistically significant reduction in headache frequency. Still, it is unlikely that the prespecified sample size would have had the power to detect a difference between the active treatment and sham.

Finally, issues with the use of sham control and identification of therapeutic gains in studies of biofeedback are important to discuss. Studies have found that the biofeedback per se does not necessarily influence treatment effect (Mullinix et al., 1978), in line with the notion that headache improvement by biofeedback is mainly driven by nonspecific effects (Autret et al., 2012). It has even been shown that instrumental conditioning in the opposite direction than what is hypothesized to lead to headache improvement—that is, hand‐cooling rather than hand‐warming—produces similar treatment effects (Scharff et al., 2002). The sham group in our study experienced a reduction in headache frequency, suggesting that the improvement in all clinical outcomes is caused by placebo and regression to the mean, and supporting the notion that there is no significant therapeutic gain (Nestoriuc & Martin, 2007). Still, the choice to conduct the study as a randomized sham‐controlled trial was mainly to evaluate the suitability and feasibility of such a sham. The fact that the sham was only a partial disruption of the biofeedback signal and that the adherence to sham in the first four weeks of treatment was high suggests that the sham signal may be “too similar” to true biofeedback, thus producing a treatment effect. This idea is further solidified by the fact that the physiological changes within and across biofeedback sessions were comparable in the verum and sham groups. With these considerations in mind, the sham intervention could be considered an active comparator and explain the small difference in treatment effects observed between the two groups.

Even though this study failed to demonstrate a convincing treatment effect of app‐based biofeedback treatment we believe there is a rationale for continued research. Firstly, the mobile setup and self‐administration allow for widespread biofeedback use. This may help overcome the limited use because of its time‐ and resource‐demanding nature. Secondly, the treatment has a significant cost benefit over traditional biofeedback. The total consumer price will likely be constituted of only a one‐time purchase of sensors (likely in the magnitude of €100–300 based on similar available technology), and no regular consultation costs. The consumer price may be even lower as the setup is easily adaptable to already existing wearables the user may have at home. Finally, the treatment has a highly beneficial AE profile. Only one case of AEs was observed, and previous studies using the same setup observed similar AE profiles (Stubberud et al., 2018, 2020). This is superior to the most commonly used prophylactic drugs, which all have several AEs in the pediatric population.

There are several measures that should be considered for future iterations and studies of the similar app‐based biofeedback treatments. The intervention should include more comprehensive instructions, guidance during biofeedback sessions, and even adjunctive therapies such as relaxation. Such features should be intelligently implemented into the app to ensure therapist‐independence and may facilitate the effect of the treatment packages observed in traditional biofeedback. In addition, measures should be taken to keep adherence high through means such as regular reminders, motivation, and gamification (Pramana et al., 2018). These measures to increase adherence could be improved through real‐time tracking of back‐end data, with customized feedback to individual users based on their performance. Next, the use of a sham control group should be carefully considered. As we experienced in this study, it is difficult to create a biofeedback sham that accurately mimics the effects of a proper placebo. A more fruitful approach might be to show noninferiority compared to the most commonly used prophylactic medications, and the study should be powered to detect small treatment effects.

### 4.3 Study limitations

The main limitation of this study is the small sample size. This has clearly reduced the precision of our estimates and limited interpretability of clinical outcomes both in the biofeedback and sham groups. Slow recruitment leading to a low sample size may be explained by the general under‐diagnosis of migraine in the pediatric population and incorrect choice of recruitment channels (Krogh et al., 2015). In addition to the small sample size, the study suffered from attrition and missing data. Several participants were excluded or declined to participate, and the overall adherence was low resulting in missing data, which further decrease confidence in our estimates.

### 4.4 Conclusion

In this study, we observed a small reduction in headache frequency in the active treatment group that was not statistically significant nor superior over sham. The limited treatment effect may in part be explained by the minimalistic nature of the intervention, low adherence rates, attrition, underpowered analyses, and an unsuited sham comparator. Still, the observed reduction in headache frequency suggests that an almost completely therapist‐independent biofeedback app may be an effective, highly tolerable and cheap treatment option, provided significant alterations to the treatment setup and study design are made. Future iterations of the intervention should include a more comprehensive intervention and ensure increased adherence through means such as gamification. Future studies of the intervention should strongly consider a noninferiority study design with an active comparison group and be powered to detect small, but clinically relevant, treatment effects.

## CONFLICTS OF INTEREST

NTNU Norwegian University of Science and Technology and St. Olavs Hospital, Trondheim University Hospital may benefit financially from the commercialization of the proposed treatment through future possible intellectual properties. This may include financial benefits to the authors of this article. Dr. Stubberud is a co‐founder of the Nordic Brain Tech AS, a spin‐off company that was established based on the proposed treatment in this and previous studies at the NTNU Norwegian University of Science and Technology. Dr. Stubberud is a co‐inventor of the proposed treatment in this study and may benefit financially from a license agreement between Nordic Brain Tech AS and NTNU Norwegian University of Science and Technology. Dr. Linde is a co‐inventor of the proposed treatment in this study and may benefit financially from a license agreement between Nordic Brain Tech AS and NTNU Norwegian University of Science and Technology. Dr. Brenner declares no potential conflicts of interest concerning the research, authorship, or publication of this article. Dr. Heier declares no potential conflicts of interest concerning the research, authorship, or publication of this article. Dr. Olsen is a co‐founder of the Nordic Brain Tech AS, a spin‐off company that was established based on the proposed treatment in this and previous studies at the NTNU Norwegian University of Science and Technology. Dr. Olsen is a co‐inventor of the proposed treatment in this study and may benefit financially from a license agreement between Nordic Brain Tech AS and NTNU Norwegian University of Science and Technology. Dr. Aamodt declares no potential conflicts of interest concerning the research, authorship, or publication of this article. Mrs. Gravdahl declares no potential conflicts of interest concerning the research, authorship, or publication of this article. Dr. Tronvik is a co‐founder of the Nordic Brain Tech AS, a spin‐off company that was established based on the proposed treatment in this and previous studies at the NTNU Norwegian University of Science and Technology. Dr. Tronvik is a co‐inventor of the proposed treatment in this study and may benefit financially from a license agreement between Nordic Brain Tech AS and NTNU Norwegian University of Science and Technology.

## AUTHOR CONTRIBUTIONS

AS: study concept, study design, data collection, data management, data analysis, interpretation of data, drafting and revision of manuscript. ML: study concept, study design, interpretation of data and revision of manuscript. EB: data collection, interpretation of data, drafting and revision of manuscript. MH: data collection, interpretation of data, drafting and revision of manuscript. AO: study concept, study design, interpretation of data and revision of manuscript. AHA: study design, interpretation of data and revision of manuscript. ET: study concept, study design, interpretation of data and revision of manuscript.

### Peer Review

The peer review history for this article is available at https://publons.com/publon/10.1002/brb3.1974.

## Data Availability

The minimum dataset required to replicate this study contains personal data and is not publicly available.
